# ReinforSec: An Automatic Generator of Synthetic Malware Samples and Denial-of-Service Attacks through Reinforcement Learning

**DOI:** 10.3390/s23031231

**Published:** 2023-01-20

**Authors:** Aldo Hernandez-Suarez, Gabriel Sanchez-Perez, Linda K. Toscano-Medina, Hector Perez-Meana, Jesus Olivares-Mercado, Jose Portillo-Portillo, Gibran Benitez-Garcia, Ana Lucila Sandoval Orozco, Luis Javier García Villalba

**Affiliations:** 1 Instituto Politecnico Nacional, ESIME Culhuacan, Mexico City 04440, Mexico; 2 Graduate School of Informatics and Engineering, The University of Electro-Communications, Tokyo 182-8585, Japan; 3 Group of Analysis, Security and Systems (GASS), Department of Software Engineering and Artificial Intelligence (DISIA), Faculty of Computer Science and Engineering, Office 431, Universidad Complutense de Madrid (UCM), 28040 Madrid, Spain

**Keywords:** malware, denial-of-service, reinforcement learning, synthetic sampling, cybersecurity, machine learning, cybersecurity datasets, artificial intelligence, q-learning

## Abstract

In recent years, cybersecurity has been strengthened through the adoption of processes, mechanisms and rapid sources of indicators of compromise in critical areas. Among the most latent challenges are the detection, classification and eradication of malware and Denial of Service Cyber-Attacks (DoS). The literature has presented different ways to obtain and evaluate malware- and DoS-cyber-attack-related instances, either from a technical point of view or by offering ready-to-use datasets. However, acquiring fresh, up-to-date samples requires an arduous process of exploration, sandbox configuration and mass storage, which may ultimately result in an unbalanced or under-represented set. Synthetic sample generation has shown that the cost associated with setting up controlled environments and time spent on sample evaluation can be reduced. Nevertheless, the process is performed when the observations already belong to a characterized set, totally detached from a real environment. In order to solve the aforementioned, this work proposes a methodology for the generation of synthetic samples of malicious Portable Executable binaries and DoS cyber-attacks. The task is performed via a Reinforcement Learning engine, which learns from a baseline of different malware families and DoS cyber-attack network properties, resulting in new, mutated and highly functional samples. Experimental results demonstrate the high adaptability of the outputs as new input datasets for different Machine Learning algorithms.

## 1. Introduction

Cybersecurity is a specialized area of IT that aims to preserve information assets in terms of integrity, confidentiality and availability. This is a key point in the technological course of human beings and tends to grow with new mechanisms, policies and procedures that provide increasingly secure ecosystems. For this reason, cybersecurity has permeated different fields of emerging technologies in society, such as health services, government, applied science, education, national security and commerce, among others. The shared objective is fundamental: the protection of information.

According to the 2021 threat report [1] presented by The European Union Agency for Cybersecurity (ENISA), there are two highly prevalent vehicles that constantly impact critical infrastructures: malware strains and Denial of Service (DoS) cyber-attacks. The first aims to compromise an information system, breaking in without authorization and achieving some benefit for the malicious actor. The second is aimed to degrade the availability of services, generally by means of a communications network. Both pose a great challenge, since, as mentioned in [2], actions and remediation must be developed more quickly and effectively in the face of more robust, intelligent and volumetric attacks.

For its part, this long race to advance defensive security has also led malicious actors to learn and refine their techniques, tactics and procedures, by building novel attack vectors [3]. Thus, there is an emerging need for asset-based analysis from an adversarial point of view, in order to propose multi-layered and holistic solutions.

However, it is difficult to categorize every latest threat and take particular solutions for granted, due to the broad landscape of cybersecurity, but, as stated in [4], there are five approaches that can be used as a basis, infrastructure security, network security, information security, cloud security and organizational security. All of them aim to serve as defensive and reactive elements in communications networks, applications, storage and in the enforcement of policies and controls, in order to establish a more effective degree of certainty in the information center, whether on-site or in the cloud.

With the above mentioned information, assessment methods can be broken down, involving firstly risk management and then cyber security assessment. Formally, the latter is a procedure that carries a technical-evaluative chain, comprising methods of penetration testing, simulation, reverse engineering, vulnerability analysis, model testing, auditing and configuration review. Thence, vulnerability identification and management has been a hot topic that has contributed to many practical state-of-the-art applications, in the areas of binary analysis, malicious activity exploration and enhancement of intrusion/prevention systems [5].

In the foreground, the focus on malware identification, remediation and mitigation has been constantly evolving, as although many solutions exist, it remains one of the most effective and progressive security threats, representing a substantial profit for malicious developers. To a large extent, it can be said that the efficiency and prevalence of malware is related to the methods for its eradication, representing a race between malicious actors and solution vendors [6]. According to a vulnerability report by McAffe [7], malware strains tend to occur in an unbalanced, but constant, manner. For example, the target sectors have been changing, as well as the attack vectors developed specific to each one. Thus, it can be mentioned that technology, education, finance, sales and government remain important categories.

Traditionally, the detection and generation of anti-malware mechanisms is a process that will involve Static Analysis (SA) or forensic post-mortem procedures, which are aimed to examine pieces of decompiled code from a binary, or in its case, a forensic analysis of network traces [8]. In addition, Dynamic Analysis (DA) is used to detail the behavior of the executable, mostly by historical activity from installation to execution and persistence, on the infected host. Both represent an initial exploratory threat unit, which essentially serves as a starting point for the creation of malware datasets, but not as unique detection mechanisms, as their limited automation and assimilation of new samples has proven to have a high impact on the generation of False Positives (FP).

On the other, DoS cyber-attacks are closely related to malware, which serve as an attack vector since some of them have the ability to control the compromised systems, such as the well-known tactics employed by worms, trojan horses or remote access programs that take control of the infected host and eventually appended it as a zombie node, to orchestrate volumetric attacks [9]. As with malware, there are purely DA solutions that correspond to defense strategies supported primarily on perimeter security devices such as firewalls or rule-based barriers activated by thresholds or load balancing rates, but, depending on the network layer, volume or type of payload, the mechanisms may not be entirely convenient, as extensive configurations are needed for rapid containment in conjunction with updated signatures for prompt detection [10].

It is well known, then, that classic solutions are feasible when the threats are already known, which include antivirus, intrusion detection & prevention systems, anomaly-based solutions and layered monitoring devices. However, for the assimilation of new patterns, the capabilities have not been sufficient and therefore, in recent years, a wide range of works have been adopted that employ, at their core, Machine Learning (ML) techniques [11].

The literature, referred to as the state-of-the-art of ML, related to malware analysis and DoS cyber-attacks encompasses different approaches from two main aspects: 1. Automate and improve the discovery and detection of new threat patterns; 2. Generate intelligent mechanisms for timely evaluation [12].

There is a vast literature of ML techniques to address the detection, classification and clustering of malware-related samples and DoS cyber-attacks, which according to the CSET (Center for Security and Emerging Technology) and the National Institute of Standards and Technology, (NIST) can be categorized depending on the scope of their function within a cybersecurity model.

Roughly speaking, the solutions proposed in the state-of-the-art mostly employ traditional Supervised Learning (SL), suggested for detection and classification of benign or malicious instances using shallow algorithms; followed by Deep Learning (DL), which is able to extend the capabilities of SL by potentially emulating the human brain and improving the desired performance; and to a lesser extent, Unsupervised Learning (UL) is used to discover new malicious patterns and cluster underlying structures; where lately, Reinforcement Learning (RL) is engaged to automate preventive mechanisms, incident response duties and active defense tasks by means of agents that learn from experience in controlled environments [13].

**Figure 1:**
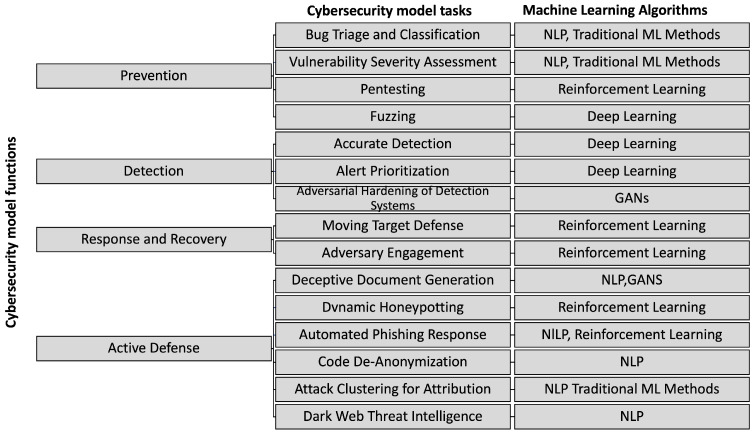
Taking into account the elements of the NIST layered security, CSET provides a model where different algorithms are applied and adapted to ML tasks, generating new opportunities for improvement in terms of prevention, detection, response & recovery and active defense. From where, GAN stands for Generative Adversarial Network - and NLP stands for Natural Language Processing.

Figure 1 describes the functions of a security model using ML, the associated tasks and the algorithms commonly applied for them.

From Figure 1 it can be summarized that the improvements that ML has offered in different branches of cybersecurity, remain in constant adaptation and evolution. However, one of the main challenges that persist in the ML landscape is the acquisition of sufficient data that can represent the desired context to be evaluated.

As discussed in [14], ML-based solutions face a major challenge in acquiring, constructing and presenting a sufficiently appropriate dataset, so that the selected algorithm can generalize the samples as well as possible. There is a debate as to whether the data should be used directly from the source from which it was obtained or whether the information should be already shaped in a tabular or a non-relational fashion. What is certain is that it is necessary to gather samples and features with the necessary dimensions, which will lead to the construction of quality data [15].

The rest of the manuscript is organized as follows: Section 2 explains the motivations behind the study of synthetic sample generation for malware and DoS cyber-attacks; Section 3 discusses the different scenarios that lead to the generation of synthetic datasets in cyber security tasks and related work; Section 4 describes the steps involved in the development of the methodology using RL; Section 5 presents and discusses the results, presenting their contribution in comparison with the state-of-the-art algorithms; finally, Section 6 provides the conclusions.

## 2. Aim and Motivation—The Problem with Cyber Threat Datasets

Problems in improper data acquisition can result in missing values, unbalanced, incomplete, high dimensionality, correlation of variables and skewed data that will obviously degrade the performance of the algorithm. This is a crucial problem in cybersecurity as poor prediction resulting from a poorly tuned algorithm can cause risky havoc around threat misidentification. Thus then, in [16], this idea can be taken up again, concluding that cybersecurity should be data- oriented with models reflecting ideal scenarios, that build intelligent models on different defensive and preventive flanks.

Cybersecurity is data, ranging from network logs to SA and DA results. This relationship between collected data, Artificial Intelligence (AI) and ML technologies results in an important instrument called the dataset, which brings together the collection of evidence needed to represent the background to be studied [17].

There are currently many ways to obtain a cybersecurity-related dataset. Specifically for malware and DoS cyber-attacks there are those already published and custom ones.

Datasets that are already publicly available come in the form of tabular information, relational and non-relational databases, operating system logs, API call sequences, raw network traffic captures, to name a few. In contrast, the customized ones focus more on obtaining information through controlled environments such as sandboxes or honeypots.

In any case, the datasets used in the state-of-the-art literature are appropriate for improving the wide range of algorithms arising from new research, but will not be able to generate new and tailored samples for explorations where more specific information is needed [18].

Although one of the ways to customize the datasets is through the synthetic generation of samples, the process is performed once the samples have already been transformed to values, this being not a true synthetic sample, but a purely statistical sampling.

This project develops a methodology called Reinforsec, which aims to synthetically generate malware and DoS cyber-attack samples, directly from an environment that most closely resembles a real attack. This, focuses on overcoming the barrier the barrier of sample acquisition, submission and evaluation, which can be quickly constructed thanks to the power of RL. The proposed agent has the ability to learn from malware PE (Portable Executable) file architecture and create from scratch a new custom dataset. Likewise, it understands DoS cyber-attack patterns at the network and application layers, simulating various types of orchestration.

The contribution of this work lies in two main branches: first, within the cyber-threat scheme, it is one of the first works focused on reducing the cost of assembling both functional raw samples and characterized sets; second, it provides a scenario in which samples can be tested with realistic sensors and observe in practical terms the outcome of synthetic mutations.

The proposal is compared with various state-of-the-art synthetic balancing and generation techniques, finally demonstrating that RL has a convenient way to create samples without expensive acquisition processes.

## 3. The Importance of Data in Cybersecurity Tasks

Currently, cybersecurity has been growing by leaps and bounds, the need for data-driven, automated and easy to interpret models has led to data science inherently converging in the development of new solutions.The scenarios have also been changing and consequently the operations of malicious actors have also been refined in order to steal their evasion capabilities. This points to the need for more efficient defense strategies that scrutinize in detail any malicious patterns that attempt to compromise an information system [19].

According to [14] the ML landscape will become more tailored to specific needs, but the critical bottleneck remains in data collection, including the appropriate identification of sample acquisition sources, data cleaning, content analysis, visualization and feature engineering. Thus, the cybersecurity area faces the same problem, but with the difference that observations are becoming increasingly difficult to detect, due to the very nature of the threat’s persistence.

Data collection is therefore not a trivial task and the availability of cybersecurity data faces a number of challenges. Some of these include the following list [20,21].

- Domain problem: obtaining samples with new behaviors is a race between early detection and the radius of impact of an unattended incident, such as zero-day incidents. While not all samples in the cybersecurity domain can be aggregated, patterns can be generated based on general behavioral policies.
- Inconsistency problems: associated with the domain problem. In an attack or compromise scenario, a data source may have many or few samples related to the incident, which may lead to some inherent problems in the data acquisition process, such as noise, incomplete, insignificant, high-dimensional and unbalanced information.
- Availability: often, due to privacy concerns, datasets will not be available for replication or testing; they may also be costly or may not contain the samples for a desired context. An important point to mention is that public disclosure of detection strategies could also be a double-edged sword, since on the one hand it refines defensive proposals, but on the other hand it gives information to the malicious actor to improve his evasion techniques.

It is difficult to address the problem of mastering and aggregating all types of datasets appropriate to each branch of cybersecurity. In [21], four categories related to such sets are maintained, around similar characteristics: attack-related; defensive artifacts; management and organizational cybersecurity datasets; and finally network and Internet macro-level datasets. The following list briefly describes the major categories of cybersecurity datasets.

- Attack-related
  - Refers to samples related to malicious intrusions such as scam, malware and web-based attacks
- Defender artifacts
  - These are samples that arise from defense system logs such as alerts, anomalous patterns and configurations.
- Management and organizational
  - It is related to behavioral data around security policies involving users, malicious actors and threats that impact the organization.
- Network and Internet macro-level data
  - Contains malicious trace samples over Local Area Network (LAN), Wireless Area Network (WLAN) and Internet networks. It is presented as information about network traffic at different layers of the Open Systems Interconnection (OSI) model.

Although the problems that give rise to inconsistent cybersecurity-oriented datasets have already been identified. There are other challenges to be addressed, which are defined below.

- Attack-related: data collection, particularly for malware analysis purposes, has become a strenuous process. In [22], it is concluded that the escalation of malicious incursions goes hand in hand with the increase of resources to build controlled analysis of malicious samples. On the one hand, SA-oriented procedures are limited in generating single-use hashes, without considering the dynamics of behavioral change. On the other hand, DA-oriented techniques only work in phases where the binary is being executed and monitored, but do not provide incremental results. Consequently, those based on heuristics are complex to set up when the threat is difficult to detect and, therefore, more in-depth generation is required. Ultimately, tasks based on anomaly detection procedures can be easily fooled when new obfuscation and cryptographic schemes are adopted in the fabrication of the malicious binary.
- Network and Internet macro-level data: there is concern about the latent increase in DoS cyber-attacks, which have eventually become a weapon for hire or sale, available to any user. With this, the variety, intensity and volume of traffic generated during an attack on the network or Internet has considerably changed the landscape of sample acquisition. This is a major challenge when replicating an attack in controlled environments, specifically for an ideal scenario of reflected DoS cyber-attacks [23].

The factors mentioned above are not the only factors that affect the generation of new samples, but also the conditions under which they were acquired, which may contain time-related failures and the complexity of their own construction process. On that premise, the generation of samples in machine emulators are good options to control the states of the system to be evaluated, but they lack of primordial semantics from the architecture of the operating system itself. Then again, first and second type hypervisors are better options as they allow isolation from the client operating system and hardware, but have difficulties in para-virtualization artifacts. Lastly, in bare-metal environments, the sample is explored in a fully realistic architecture, but it cannot be scalable in terms of resources, and, moreover, the appropriate circumstances do not exist for it to be restarted to previous states, thus losing an analysis already started [24].

One of the alternatives, given the complexity of generating sufficient data, are synthetic samples, which aim to replicate distributions of an original set, sticking as closely as possible to the domain of the set [25]. Among the techniques for generating synthetic samples are those described in Table 1.

In contrast to what is shown in Table 1, the application of RL techniques has demonstrated a less complex way of generating synthetic samples, with the main objective of bringing them as close as possible to those acquired from the source, with an ideal balance of the characteristics of the samples [26]. One of the most compelling examples in the use of synthetic samples, which sufficiently resemble a real ecosystem, is Health-Gym [27], a tool for producing synthetic data, related to medical records. By testing correlations with real data and a RL agent, the results demonstrated that synthetic samples can be effectively used to follow up diseases such as Human Immunodeficiency Virus (HIV), Acute Hypotension and Sepsis. The authors demonstrated that by means of statistical tests, mainly the Kolmogorov–Smirnov (KS) test, it is possible to measure the variation and similarity of synthetic towards real data, from which the RL outperformed those derived from GANs as a function of static correlation. Motivated by these broad scopes, this project will address the principles of RL, focusing on complex samples to be generated in controlled environments, such as those that provide network and client-side sandboxes for malware and DoS cyber-attacks.

## 4. Proposed Methodology

To carry out the generation of synthetic samples from a real source, two environments controlled by RL were prepared: the first one for malware samples, further described in Section 4.1 and the second one for DoS cyber-attack samples, as detailed in Section 4.3. This section describes the main concepts that give life to the proposed ecosystem [28].

### 4.1. Reinforcement Learning for Creating Synthetic Sample Gyms

Reinforcement Learning is a branch of responsive Artificial Intelligence, based on interaction-oriented generalization, in which an agent achieves learning through interaction with a particular and controlled environment. In this type of training, the agent is not taught what actions to take, but rather the agent learns as a consequence of the behavior it takes, since each interaction will be tailored by a given penalty or reward.

This project applies the OpenAI Gym [29] library of the Python programming language, which employs an agent manipulated by a Partially Observable Markov Decision Process (POMDP) [30]. The agent is placed in a gym, which is defined as an environment where the learning context is abstracted and where the agent can observe the ecosystem, receive rewards and complete its goal.

In its most basic form, the main OpenAI-RL component consists of an agent *a*, which is controlled by an algorithm designed to learn from states $s_{t}$ within an ecosystem or environment, given a time function *t*.

In order to generalize the observation of the environment, a Markov algorithm induces the agent *a*, to interact with the environment on a discrete time scale *t*, from which the following elements {S,A,T,R,Φ} are derived; where *S* is the state space, *A* is the set of actions in a given discrete space, $P\left(s_{t+1},s_{t},A_{t}\right)=p\left(s_{t}t+1,s_{t},A_{t}\right)$ is the transition function that measures the probability of obtaining the next state $s_{t+1}$, from an initial state, where at each step an immediate reward $R(s_{t},A_{t})\to R$ is given; and consequently, Φ∈[0,1) is the penalty factor, in case such action is not fulfilled.

RL also takes a crucial step, called state policies γ, which maximizes the probability that an action $A_{t}$, in a state $s_{t}$, can be considered as the most appropriate. Like classical algorithms, such a function presents an optimal degree of transition, where the reward is expected (E) to be maximized and the penalty minimized, as shown in Equation (1).

(1) $$J\left(\gamma \right)=E\left[\sum_{t=0}^{\infty }\Phi^{t}R\left(s_{t},A_{t}\right)\right]$$

However, in a real RL environment, agent *a* may degrade its learning potential and not fully observe the whole panorama, so POMDP, adds an element of belief that allows it to still partially observe the system and learn with the necessary actions in each state. Therefore, POMDP is defined as a tuple {S,A,P,R,O,Z,Φ}, where *S*, *A*, *P* and *R* are the already defined transition state elements; *O* is the observation space; *Z* is a set of observation probabilities and Φ is the penalty value.

The main advantage is that a partial observation gives a preliminary overview of the environment. Thus, in each period *t* the agent chooses an action $A_{t}$ that causes a transition to a state $s_{t+1}$ with probability $P(s_{t+1}|s_{t},A_{t})$, which receives an observation *O* that depends on the new state of the environment with probability $Z(O|s_{t},A_{t})$. In this way, the agent will be able to transit while understanding the change of the environment, and induced to obtain the reward $R_{t}$. The process is iterative, so that the agent *a* will have to select which policies γ maximize the rewards at each time *t*. Equation (1), can be reformulated as a maximal expectation, as shown in Equation (2).

(2) $$J\left(\gamma \right)=\max E\left[\sum_{t=0}^{\infty }\Phi^{t}R\left(s_{t},A_{t}\right)\right]$$

Despite the enhancement achieved by the POMDP algorithm, the scope of a policy could increase the complexity of the partial observation procedure, degrading the search for the most optimal one. To follow up on this, Q-Learning (Quality Learning) approach adds a low-cost procedure to the search for adequately improved policies, also called out-of-policy control progression [31]. The transition state of Q-learning is defined in Equation (3).

(3) $$Q\left(s_{t},A_{t}\right)\leftarrow Q\left(s_{t},A_{t}\right)+\alpha [R\left(s_{t+1},A_{t+1}\right)+\Phi \max Q\left(s_{t+1}+A_{t+1}\right)-Q\left(s_{t},A_{t}\right)],$$

where *Q* is the transition estimation function to a state $q_{*}\left(s,A\right)$ and α is the parameter to converge the cumulative learning radius in the algorithm.

### 4.2. Creation of Synthetic Malware Samples in PE Formats by RL

There are numerous ways to analyze a malicious object, mainly those based on objectives, such as detection, similarity analysis and generation of new taxonomies, which involve a more targeted scope in crafting new variants, families, likeness and differences in synthetic samples [6]. In extension to this, more particular ways can be reproduced, focusing on feature extraction, involving the already known SA, DA and Hybrid Analysis; where byte sequence calls, system calls, opcodes, network activity, file system changes, CPU registers and PE analysis are synthesized [32].

Nevertheless, the method based on PEs is one of the most widely used for the subsequent triaging of malicious objects, generally within the Windows operating system. This file format is currently supported by the Intel instruction set, Advanced Micro Devices (AMD) and some Advanced RISC Machines (AMR) variants. The great advantage of adopting this analysis mechanism is that a PE can provide sufficient information about the objective of the binary and the activity performed within the operating system during its execution [33].

In addition, these properties are coupled with the flexible structure of the PE, which allows preserving a common format in different versions of the operating system, increasing the ability to obtain information related to its mutations or polymorphism. [34].

According to [35], the intrinsic value of the PE file lies in part with the metadata that converge in the structure of the binary. With this, values related to code architecture, timestamps, memory regions and other flags can be explored. Among the most common examinations are those centralized on the file header, detailing the file signature and components that lead to execution, and those that deal with the optional header, describing specific operating system fields, data directories and binary-specific domains.

The PE file is basically made up of two sections, the headers and the body. These two are further subdivided into more sub-sections-conforming structured headers, aimed to link the necessary information for the operating system loader to execute a file.

**Figure 2:**
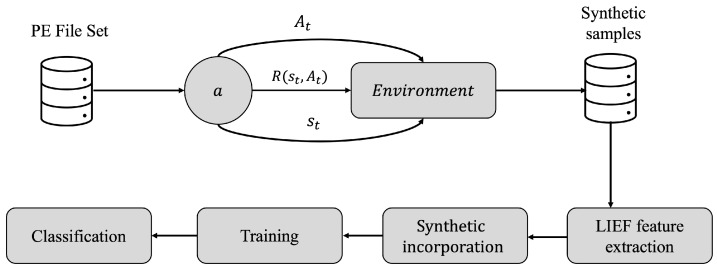
Proposed framework for the generation of synthetic binary samples in PE format using RL.

In order to perform the sample mutation process using RL, the base agent developed in [36] was taken into account. The manipulation of the file is set up in a black box environment with a supervised policy (Λ), which will set the criteria for the mutation to be sufficiently generalizable, so that the binary can be classified as malicious. Figure 2 shows the framework for generating synthetic samples from mutated PE format files using RL.

First, the samples are obtained from different online malware repositories; then each one is submitted as an individual environment, where the agent *a*, will learn from a series of actions $A_{t+n}\in A;\forall n\in Z^{+}$ in a series of steps, in transition states $s_{t+n}\in S;\forall n\in Z^{+}$, with a Λ policy established to evaluate if the sample is successfully mutated, where the series of rewards $R\left(s_{t},A_{t}\right),\cdots ,R\left(s_{t+n},A_{t+n}\right)$ will be proportional to the search of successful actions by a function *Q*; once the mutation is fulfilled, the sample is then grammatically instrumented using a PE header extractor called Library to Instrument Executable Formats (LIEF), in order to analyze whether the synthetic sample is functional, so that it is finally added to the final set. As a concluding step, the samples are trained and subjected to classification criteria by different algorithms to demonstrate the performance of the samples. In Section 4.2.1, Section 4.2.2 and Section 4.2.3 the above-mentioned procedure is detailed in depth.

#### 4.2.1. Data Collection

It is complex to establish a criterion indicating which categories or families of malware are suitable for obtaining raw binary samples. In fact [37], it is concluded that leading managers such as VirusTotal [38] or VirusShare have a high incidence of sample mislabeling once sensors have detected an object as malicious.

Sample duplication is also a negative effect at the time of selection of malware base samples, thus the bias directly impacts the generalization of a classification problem by increasing the redundancy factor, which is a ratio that measures the number of samples similar enough to be considered the same [39].

One of the basic reference points for gathering observations of malicious objects is the Malware Reference Dataset with Ground Truth Family Labels (MOTIF) [40], a file-oriented database in PE format, where 454 different families distributed over 3905 samples are presented. With that, the unique hashes identifying each one can be taken and the binaries downloaded in a controlled environment.

In addition, a well-known sample downloading framework called Endgame Malware Benchmark for Research (EMBER) [41], which groups malicious and benign PE files, was studied. This allows us to obtain a dataset free of legal or security implications. In this dataset, each sample is published along with its unique hash, and a label revealing whether the file is considered malicious or benign.

#### 4.2.2. Learning Space: Actions and States

To initiate the RL environment, it is necessary to establish the conditions of the reward function in each state $R\left(s_{t},A_{t}\right),R\left(s_{t+1},A_{t+1}\right),\cdots ,R\left(s_{t+n},A_{t+n}\right)$. OpenAI Gym provides the option of integrating a supervised learning algorithm *C* that works as a control state and evaluates the certainty of compliance with policy f:Q→Λ. This will determine whether the modified sample is detected as malicious, otherwise, the agent *a* will be penalized by the discount function Φ.

The action space A∪S is defined as the set of actions *A*, which are combined with the state space *S* and will represent an available mutation, to create a new synthetic sample, without changing the functioning of the same. For this, the authors in [42] define a series of categories that can be taken into account for binary evasion techniques and that can be considered for the generation of mutated samples: the metadata of the PE header, the metadata corresponding to the sections that structure the PE (section name, size and characteristics), the metadata of the import/export table, the count of readable strings and the byte histogram.

According to [43], modifications to a PE file are directly associated with evasion techniques, tactics and procedures, which allow to strengthen persistence within a host. The elements considered to establish the Λ policy for mutations are listed below.

- Adding an obfuscated function to the import table
- Manipulate the common name of the sections in each offset
- Build and increase the spacing of the format sections
- Add bytes to the remaining free space at the end of the sections
- Create a new entry point that immediately goes to the original entry point of each offset
- Remove information about compilation and debugging signatures
- Package the binary and add bytes at the end of the last section of the PE file

To illustrate the process of learning and generating new modified synthetic samples, Algorithm 1 describes the steps of this concept, where Λ is the policy that establishes the set of possible mutations.

As mentioned before, the policy Λ is composed of two main elements: first the mutation elements $\{\lambda_{1},\cdots ,\lambda_{k}\}\in \Lambda$, comprising the possible interventions on the file, and second, the supervised algorithm *C*, which will serve as a control state. Therefore, the policy will obtain in a time *t* a reward *R* if the mutated file is sufficiently generalizable as malware $Q\to R(s_{t+1},A_{t+1})↔C=1$, given a successful search for actions *A* in the *Q* function, or, will be classified as non-malware, where the discount factor will be applied Q→Φ↔C=0,∀Φ∈R.
**Algorithm 1** Learning process, out-of-policy**Require:**$Q(s_{t},A_{t})$, $\forall s_{t}\in S,\forall A_{t}\in A$ arbitrarilty, and Q(terminalstate,·)=0    **for each** $s_{t}$ **do**    **Initialize** agent *a* with sates *s* at time t+1        **for each** $s_{t+1}$ **do**          Choose *A* from *S* using Λ derived from *Q*          Take action $A_{t}$, observe *R*, $s_{t+1}$          $Q\left(s_{t},A_{t}\right)\leftarrow Q\left(s_{t},A_{t}\right)+\alpha [R\left(s_{t+1},A_{t+1}\right)+\Phi \max Q\left(s_{t+1}+A_{t+1}\right)-Q\left(s_{t},A_{t}\right)]$          $s_{t}\leftarrow s_{t+1}$        **end for**    until $s_{t}$ is terminal, hence the PE is fully mutated    **end for**


Likewise, the arbitrary state allows to start with a random set of manipulations encompassed in Λ for each transition step $s_{t+1},\cdots ,s_{t+n}$, achieving a successful search for at least one mutation, by means of the *Q* function. Likewise, if the agent does not find an action *A* in a time *t*, the environment is discarded and continues iterating with the next sample, reaching a terminal state, when there are no more rules in the policy and environments to pursue.

#### 4.2.3. Synthetic Header Checking and LIEF Feature Extraction

Synthetic samples must ensure stability and an optimal action search, so the mutation must be subjected to a grammatical analysis process, to test that it is sufficient to be able to run as a binary in the operating system.

To examine the quality of the mutated synthetic sample, LIEF [44], a library for instrumenting files in PE format, was disposed to grammatically parse the binary and test whether modifications of each section are functionally adequate during execution.

**Figure 3:**
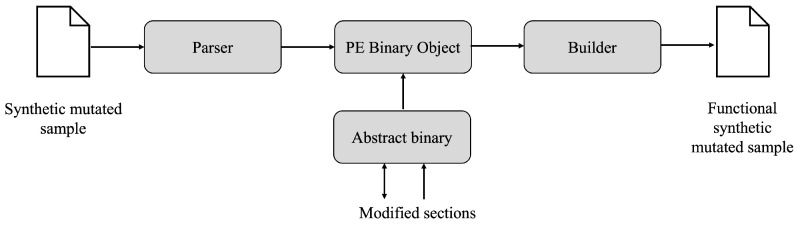
Instrumentation components of the synthetic sample in functional form.

Figure 3 outlines the process of functional analysis of a mutated synthetic sample using LIEF instrumentation.

The process consists of submitting the sample to a parser and a low-level packer, better known as a builder. The former decomposes each of the modified sections that make up the abstract binary in terms of content and size; the builder then functionally analyzes the execution of the file to check that it is valid and free of environment or execution errors. Lastly, the builder produces a synthetic sample in the form of a raw binary, after deciding whether it passes or not the functionality tests.

Indeed, fresh synthetic samples eventually go through a feature extraction and transformation technique, so that it can be used by SL algorithms. Hereby, with EMBER all data were standardized in order to achieve a proper feature representation, and, thus achieve a sufficiently homogenized format [45].

The EMBER structure is sketched by a collection of documents in JSON format, from which eight different groups of parsed and formatted features are distributed, as stated in Table 2.

From Table 2, it can be observed that there is a wide variety of data types, shapes and dimensions, in terms of features. Classical transformations, e.g., from unstructured data to vectors by imputation, or from text strings to weight vectors, usually increase the size of the feature set, causing under- or over-fitting effects [46].

One of the most effective approaches to unstructured feature representation is hash mapping, which reduces dimensions and transforms the features into an index vector that links the original feature value with its associated hash [47].

The main idea is to transform the values of Table 2, into *f* finite elements for each feature *F*. Then, each set *F* must be coupled to a fixed size vector, constructing a hashing function $\phi :F\to R^{n}$, and a sign function ζ:F→{−1,+1}. In this way, it can define the tuple of feature hashing, as described in Equation (4).

(4) $$\phi \left(f\right)=\zeta \left(f\right)v_{h(f)},$$

where *v* is the unit vector, of which each value *f* is mapped and h(f) is the computation of the bijection of each index and the linked feature *f*, i.e., h:F→{1,⋯,n}.

In total, 11,000 synthetic samples were built, of which 8000 were labeled as malware (class 1) and the remaining 3000 as benignware (class 0). Each, was stored in the EMBER format with an integrity hash, representing the sample identifier.

### 4.3. Synthetic Sample Generation of DoS Cyber-Attacks Using RL

In terms of DoS cyber-attack analysis, the main task is to determine what type of category is critical to simulate, and with this, to consider what will be the most appropriate features within the network, transport and application layers.

Traditionally, a DoS cyber-attack is explored according to the vulnerability to be exploited: flooding attacks, application attacks, protocol exploitation and malformed packet attacks [48]. The first sends large volumes of traffic to the target system to congest bandwidth, saturating the response on the victim’s side; the second exploits the IP protocol addressing function in network devices to amplify and reflect a payload and send messages to all stored addresses, considerably reducing bandwidth; the third is based on the exploitation of specific features within a protocol to consume excessive amounts in its messaging process, exhausting its resources; and the last one reconstructs random high-length messages in the IP protocol, specifically in the address and packet headers, to collapse the target’s information reading process, degrading its resources [49].

In general, the traceability of a DoS cyber-attack is summarized in applications that can provide a log based on the responses of signatures or alerts caused by an anomalous event. In [50], it is concluded that most of the evidences that can be useful to examine such attack are those based on anomalies, since they allow observing those patterns that deviate from a base profile.

Having described the above, gathering DoS cyber-attack samples has become an arduous and difficult task, as it requires a network node where the variety and occurrence of the attack has a high chance of being observed, as well as a sensor capable of capturing a high volume of traffic.

In [51], it has been proposed that to overcome the limitations of capturing high volume traffic resulting from DoS cyber-attacks, simulated senors can be adopted in controlled conditions. However, in the end, whether in real or virtual environments, the meeting point will always be a network interface in listening mode that adequately supports a large number of frames and thus a tool that can sense and store them.

Among the most widely used network traffic capture and storage tools, wireshark and tshark can be mentioned. These have the ability to segment the frames and packets, so that they can be presented in numerical or categorical terms, which will lead to the creation and characterization of a set of data [52].

Once the network capture file is built, you can begin to identify candidate features, especially those that are composed of real values, such as: the packet length within the bandwidth, the average length of a sending and replying packet, the number of packets, average time intervals and numerical representation of protocols used. On the other hand, categorical characteristics are mostly based on the application layer, where the payload is suitable as a textual corpus, message sequences or graphs [53].

**Figure 4:**
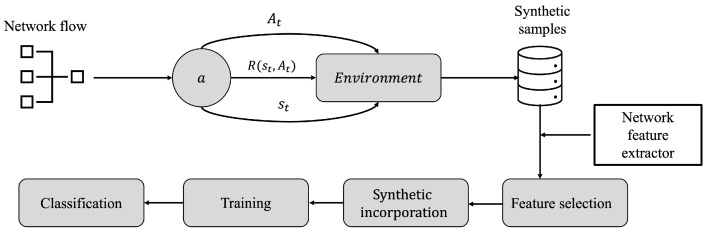
Proposed framework for the generation of synthetic samples of DoS cyber-attacks employing RL.

Considering the complexity and limitations of other techniques for the capture and generation of DoS cyber-attack samples, this project takes into account a set of input data of network flows, previously captured and packaged in Packet Capture (PCAP) format, which will serve as a baseline to train an RL algorithm and produce new synthetic samples. Figure 4 depicts the workflow for the generation of synthetic samples for the construction of a set of DoS cyber-attacks. In Section 4.3.1 and Section 4.3.2, the proposed model is discussed in more detail.

First, the network flow replays a previously captured dataset with frames containing different DoS cyber-attacks samples embedded mainly in TCP (Transmission Control Protocol) protocol; then, the samples are subjected to a RL environment where the agent *a* is aimed to learn from the properties of each frame based on network characteristics, namely, the mutation actions $A_{t+n}\in A;\forall n\in Z^{+}$, subjected to a series of transition steps $s_{t+n}$ and parameterized to a Lambda policy of a *Q* quality function, which is finally evaluated, based on a series of $R\left(s_{t},A_{t}\right),\cdots ,R\left(s_{t+n},A_{t+n}\right)$ rewards.

#### 4.3.1. Data Collection

Within the DoS cyber-attack literature, different datasets have been exposed, which are mostly the result of capturing network sensors in PCAP format. Even so, in this project it was necessary to reconcile a significant proportion of samples that reflect, variety and size. The state-of-the-art [48,54,55,56,57] has provided an important selection of them, which were examined in terms of their content.

The aforementioned study showed that although many datasets provide relevant information to initiate a baseline sample selection, there are problems of incomplete capture, missing data or lack of diversity and heterogeneity. With this, two data sets were selected that broadly summarize the variety of DoS cyber-attacks, in conjunction with regular network traffic:

- CICDDoS2019 [58]: contains benign network traffic and distributed DoS cyber-attacks via SNMP Simple Network Management Protocol (SNMP) reflected attacks, NetBIOS reflected and timestamp attacks, Lightweight Directory Access Protocol (LDAP) amplification attacks, Trivial File Transfer Protocol (TFTP) amplification attacks, Network Time Protocol (NTP) amplification attacks, Synchronize (SYN) flooding attacks, WebDDoS Hypertext Transfer Protocol (HTTP) specific-protocol attack, Microsoft SQL Server (MSSQL) specific-protocol attacks, User Datagram Protocol (UDP) lag flooding attacks, Domain Name System (DNS) flooding attacks, and Simple Service Discovery Protocol (SSDP) reflection attacks.
- Customized set: for application layer DoS cyber-attacks, hulk and slowlowris protocol-specific flooding tools [59] were deployed. To build the scenario, 12 virtual machines were configured with Windows operating system versions 7 and 10 (half of each pool of virtual machines), mounted on 4 PCs with 16GB of RAM, Intel core i7 processor and Ubuntu 18.0.42 operating system. The series of attacks were orchestrated towards a gateway with permissive rules (any-any) and also a spare port was configured in the generic switch to capture traffic on a third computer with Ubuntu 18.02 operating system and tcmpdump as a logging tool.

In total, with the CICDDDoS2019 data and the custom set, 58,641 samples were congregated of which the following percentages are presented based on the type of DoS attack: specific protocol attacks at the application layer (WebDDoS, hulk, slowlorwris), specifically HTTP 35% and MSSQL 5%, flooding (SYN, DNS, UDP) 25%, amplification (LDAP, TFTP, NTP) 15% and reflection (SNMP, NetBios, SSDP) 20%.

#### 4.3.2. Learning Space: Actions and States

According to [60], the generation of synthetic samples of network traffic is a process that addresses two types of levels: functional, which depend on frame and packet level injection to make reproduction as realistic as possible; and non-functional, which depend on the scalability and quality of the synthetic sample. To address the DoS cyber-attack network traffic mutations, a series of properties are considered as a baseline to construct new features that will be used during the RL training. The aforementioned is described in Table 3.

As stated in [61], the properties derived from Table 3 can be decomposed into specific values that encompass the network, transport and application layers. Table 4 then, describes those that can be used for mutations and future generation of DoS cyber-attacks synthetic samples.

Once the mutable features have been identified, it is necessary to submit agent *a* to the training process, where the environment will be each frame to be assessed, within the replay of the PCAP files. For this, an Open-AI Gym model called Gym-DoS was configured, which allows linking each network traffic flow to the set of actions within a space A∪S, that will modify each feature/property in a series of $\left\{s_{t},s_{t+1},\cdots ,s_{t+n}\right\}$ transitions, under the constraint of the mutation elements $\left\{\lambda_{k},\cdots ,\lambda_{k}\right\}$ of the Λ policy derived from the quality function *Q*.

The learning algorithm is associated with the one presented in Section 4.2.2, but, unlike malware mutations, in a network flow the mutations must be executed within the start of each conversation. This is because within PCAP captures, each frame arrives at the network interface indiscriminately, regardless of the protocol used. In order to modify the entire conversation, the following must first be identified for each protocol: in TCP-IP protocols the three-way handshake, in UDP protocol the streaming of the first datagram flow and for the application layer, the specific protocol as well as the start and end buffers for sending the payload. Algorithm 2 shows the learning process for a DoS cyber-attack sample mutation.
**Algorithm 2** Learning process, out-of-policy for mutating DoS cyber-attacks frames**Require:**$Q(s_{t},A_{t})$, $\forall s_{t}\in S,\forall A_{t}\in A$ arbitrarilty, and Q(terminalstate,·)=0    **for each** network frame **do**    Sort the start of the protocol conversation    Build an action space *S* for each frame        **for each** $s_{t}$ **do**    **Initialize** agent *a* with sates *s* at time t+1            **for each** $s_{t+1}$ **do**          Choose *A* from *S* using Λ derived from *Q*          Take action $A_{t}$, observe *R*, $s_{t+1}$          $Q\left(s_{t},A_{t}\right)\leftarrow Q\left(s_{t},A_{t}\right)+\alpha [R\left(s_{t+1},A_{t+1}\right)+\Phi \max Q\left(s_{t+1}+A_{t+1}\right)-Q\left(s_{t},A_{t}\right)]$          $s_{t}\leftarrow s_{t+1}$            **end for**    until $s_{t}$ is terminal, hence the DoS cyber-attack frame is fully mutated.    **end for** **end for**
  

With the new mutated synthetic samples, the same evaluation procedure of Section 4.2.2 is reproduced; where a supervised algorithm *C* controls the veracity of the sample, as a state control, estimating that it is a malicious DoS cyber-attack sample, so the reward $R(s_{t+1},A_{t+1})↔C=1$ is granted; otherwise, if C=0, the discount factor Q→Φ↔C=0,∀Φ∈R is applied.

In due course, each synthetic DoS cyber-attack sample was transformed to real values using the FlowMeter technique [62], which transforms the network flow data, from PCAP format to statistical information, allowing the extraction of 79 features.

The above process is detailed in [63], where it is summarized that statistical values of network traffic are a great option to represent anomaly flows, especially in scenarios where it is desired to apply SL to classify malicious and benign traffic. However, when performing the first data presentation, the features that depend on MSS and Windows size, presented a high variance, which, evidently could bias its application with ML algorithms.

To resolve the effects of variance, a process for feature selection and extraction was executed, using the Principal Component Analysis (PCA) algorithm [64]. Table 5 depicts the features that have been shown to have the best variability of the DoS cyber-attack dataset.

In total, 50,000 samples were obtained from DoS cyber-attacks and another 50,000 from benign traffic, mainly from TCP protocol, regular DNS requests, browsing to web sites and APIs, telnet connections, FTP data upload and Secure shell (SSH) data transfer.

## 5. Results and Discussions

This section considers two scopes to present the results obtained for the generation of synthetic malware samples in PE format and DoS cyber-attacks samples.

- First scope: the samples are subjected to traditional detection tools before being transformed: for synthetic malware samples, VirusTotal [65] is used as a sensor for different antivirus programs; for DoS cyber-attacks samples, the generic detection CloudShark [66] firewall based on behavioral signatures is used.
- Second scope: the already characterized samples are compared with different synthetic generation and balancing techniques and finally evaluated in terms of performance metrics by different state-of-the-art SL algorithms.

Table 6 shows the detection radius with synthetic samples before characterization.

The following can be deduced from Table 6:

- VirusTotal: the sensor works by assembling different antivirus machines, which together determine the evaluation criteria of the submitted sample. The accuracy value is taken into account as follows: 40 to 60 independent machines are used to perform a diagnosis of the object by means of a simple triaging, if 60% exceeds the malicious assignment, the object is considered as such, otherwise it is considered clean.
  Although it is one of the main early malware evaluation mechanisms, it is difficult to summarize the specific category of the synthetic samples. Even so, it could be observed that in the labels of each machine, 40% presented a signature denominated as Generic, 31% as Malicious, 13% as Trojan, 8% as Riskware, 3% as Adware, and, the rest in different taxonomies.
- CloudShark: the tool allows loading PCAP files and analyzing the degree of threat contained in the sample. It is worth mentioning that each of them can be composed of different frames, called streams, i.e., the sample contains the entire conversation. To determine whether the conversation presents any anomalous pattern, CloudShark compares the IDS Snort signature and returns a categorical value of the threat as low, medium or high.

From the above, it was observed that the most common rule is Malware Other with 63% prevalence, Potentially Bad Traffic with 26%, Potential Corporate Privacy Violation 4% and Unknown traffic, the rest.

On the other hand, in order to present comparative results from the point of view of algorithms and synthetic sample generation techniques, Table 7 presents the related works, as well as the most outstanding algorithms for the evaluation of malware samples and DoS cyber-attacks.

Of the techniques presented in Table 7,^60^,^67^,^68^,^69^,^70^,^72^,^74^] perform mutations outside the ecosystem, when the samples have already been processed, extracted or transformed to some data representation, whether from malware or DoS cyber-attacks. In [73], the authors present a more realistic approach, as the focus is on mutations of statistical values that resemble those of a network attack such as DoS. Nevertheless, the mutations occur only in DoS cyber-attacks on the application layer and the evaluation metrics are based mainly on the times of attack duration and maintenance of entropy percentages, leaving aside other important values such as payload, the variety of IP addresses, ports and volume of conversations.

Regarding the evaluation of synthetic sample generation processes used for malware and DoS cyber-attacks, the state-of-the-art works [60,67,68,69,70,71,72,73,74] employed different SL and DL algorithms, as shown below:

- Shallow algorithms: are those that the literature refers to as classical, where learning takes place by means of predefined features and labels, in a continuous forward model. The following algorithms derived from Table 7 were reported: Multi-Layer Perception (MLP) [67,74], Decision Trees (DT) [67,74], Logistic Regression (LR) [67,71], Support Vector Machines (SVM) [67,69,71], Random Forest (RF) [67,71], and Gradient Boosting (GB) [73].
- Deep learning algorithms: those based on neural networks, which perform operations on different layers that represent a simplified form of information to each of them. They are known to work with different information input and output structures. In this sense, the following algorithms were reported: Deep Residual Network (CNN+DRN) also known as ResNet-18, [68], MDGAN (the discriminator itself worked as a classifier) [70] and Feed Forward Neural Network (FFNN) [72].

It is worth mentioning that not all of the related works presented performance metrics that could be taken into account to compare this project. For this reason, only the Precision was taken as the only preponderant measure, which is expressed in Equation (5).

(5) $$\mathrm{Precision}=\frac{TP}{TP\;+\;FP}$$

It is important to note that the target class 1 represents malware or a DoS cyber-attack observation, therefore TP represents the True Positives and FP the False Positives. Analogously, the non-target class 0 represents the benignware or clean traffic, of which TN is identified as the True Negatives and FN as the False Negatives, respectively.

In addition, there is not enough information to determine most of the hyper-parameters used in the algorithms of the aforementioned works. Taking this into account, it was decided to train the samples generated with RL with the proposals of the state-of-the-art, using default parameters [75], as shown in Table 8. In the same way, it was decided to split, both, for malware samples and DoS cyber-attacks, a training set $X_{T}$ with 80% of the samples, a validation set $X_{V}\in X_{T}$ with 10 folds for k-fold-cross-validation and a testing $X_{P}$ set of 20% of the remaining samples. For $X_{T}$ and $X_{P}$, the selection of observations is random and without replacement.

From the scopes presented in [60,70], it was not possible to obtain enough information on how to reproduce the steps of the algorithm. In [70], it is shown that the GAN discriminator behaves as a classifier, but no further details are given on how to implement it. On the other hand, in [60], only statistical details of the modification of the network flows are shown, presented by the average number of packets, time windows and accumulation of data and entropy of the source-destination addresses and the TTL; lastly, such work does not include values of predictions over SL algorithms.

To present the progress of this work (RL) for the generation of synthetic samples, Table 9 addresses the results in terms of the Precision metric, with what has been obtained with related work.

From the results displayed in Table 9 the following can be observed:

- For the synthetic malware generation scope
  - GAN-MLP [67] exceeded the precision record with 99.46% towards the counterpart of this project (MLP-RL) with 99.12%. The variation is not high, however, GAN-MLP can result an unsatisfactory procedure if the number of malware samples increase, this has already been reported in [76] where the complexity is proportional to the number of inputs, making the latter unstable and slow, which could produce samples with low quality. Moreover, if the network produced by the GAN is linked as input to a MLP the number of parameters to be estimated can be exponential, generating a redundant model or with low efficiency.
  - In the matter of Fuzzy-SMOTE+ SVM [69], the precision value of 99.02% exceeded this work, compared with RL + SVM, which only achieved a percentage of 99.81%. It is worth mentioning that the Fuzzy-SMOTE computation could work as long as it is desired to report a preliminary range in the sample balancing. Even so, such algorithm only creates replicates with little mutation, that could, in the worst case, generate an overfitting phenomenon.
  - On the other hand, the shallow algorithms, trained with this scope, namely, RL + DT, RL + RF and RL + LR, obtained better results in terms of precision, with 99.71%, 99.81% and 99.45%, respectively, compared to their GAN-oriented [67] counterparts, which obtained a precision score as follows: GAN + DT with 90.43%, GAN + RF with 98.87% and GAN + LR with 96.71%.
    It is known that pure or ensemble DT algorithms are ideal for their ability to understand and interpret problems in a timely manner with little data preparation. This can be enhanced if random splitting methods such as RF are applied. However, the risk of combining trees and GANs is the instability of the model when the number of samples increases considerably, which, unlike RL the process concentrates within the malware sample mutation on the agent’s policies and not on the competition between generators and discriminators.
  - Moreover, as for shallow algorithms employing MDM [71] and One-hot-encoding, lower values were obtained as a function of the aforementioned precision, these include MDM + One-Hot-Encoding + SVM with 97.30%, MDM + One-Hot-Encoding + LR with 98.10% and MDM + One-Hot-Encoding + RF with 98.20%, which, compared to this project, the following values were obtained: RL + SVM 98.70%, RL + LR 99.45% and RL + RF 99.81%.
    This has an important reason and lies in the fact that MDM is the theoretical basis of RL, but oriented to infer the total probability between states, without considering the instability risks of adding partial observations and out-of-policy state controls, which, the present work applied with Q-learning. In addition, MDM has been shown to have problems in the optimal search for policies in transition states, when the number of samples increases and that coupled with One-hot-encoding-type characterizations, would result in identical synthetic malware samples, with little context and poor semantics.
    When MDM is added to an AWGCN [71] it is possible to obtain a scope that other neural networks cannot reach, especially when it is desirable to work with s non-structured data, particularly because of the attention layer provided. However, with large-scale data there is the possibility of suffering from noise, scalability disturbances and adverse discrepancy between the rules of the AWGCN trees, which would require sufficient mini-batch tasks to approach a high-volume environment. The results demonstrated in terms of precision showed that MDM + AWGCN + SVM with 99.20%, MDM + AWGCN + LR with 99.30% and MDM + AWGCN + RF 98.70% in percentage, obtained lower values than this project with RL + SVM 98.70%, RL + LR 99.45% and RL + RF 99.81% values.
  - By comparing hybrid GAN algorithms such as DCGAN + ResNet-18 [68] and MDGAN [70], it can be observed that different types of malware features such as pixel representation and sequences of APIs can be combined and then the resulting matrix can be sampled at low cost, reducing the training time and synthetic production. However, in addition to the already-mentioned disadvantages of GAN, a high volume of inputs can affect the calculation of probabilistic distribution when combining two or more DL mechanisms. In summary, both DCGAN + ResNet-18 and MDGAN obtained lower precision values than proposed in this project with RL, with 90.00% and 95.90%, respectively.
- For the scope of DoS cyber-attacks
  - The scope presented in [72], Statistical learning + FFNN, has an interesting potential since mutations are fully adequate to descriptive statistics of different samples. However, there are not enough indicators of sample quality, volume and functionality. Moreover, a FFNN is known to present many parameter fitting problems, especially since its optimization function is based on gradient optimization. Even so, when testing this RL + FFNN project, better results were obtained, in terms of performance with 96.41% compared to its counterpart with a value of 88.00%.
  - Although in [73] the GAN is stabilized with the GP penalty algorithm, to minimize boosting and gradient vanishing effects, the WGAN is not immune to high volume impact during training phases, in fact, the penalty avoids the cost of hyper-parameter computation. Indeed WGAN, presents oscillation and learning convergence problems for new samples, especially those of large content such as DoS cyber-attacks. The SL part of this scope employed the GB algorithm, despite that, no relevant data were available to compare the precision metric of the algorithm. However, it was possible to calculate the Area Under the Receiver Operating Characteristic (ROC) Curve (AUC), which measures the performance of the model at different thresholds. The AUC is then said to be measured in an interval of (0,1), where 1 is a perfect model. This indicates that the RL + GB model of this project also obtained a better result than GP-WGANs + GB, with an AUC of 0.87.
  - The main limitations of MDM have already been mentioned. In [74], it was explored to use it with PRISM, in order to stabilize states that could result in unpredictable behavior. A problem that could occur in this case is that, being a simulation algorithm, the costs generated per se would be exhaustive and it could not be guaranteed that the samples would result in sufficient quality. In fact, RL, avoids this scenario by reducing the unfavorable states, so that the agent learns what is necessary and reduces the search for optimization criteria to increase. Consequently, both MDM + PRISM + MLP and MDM + PRISM + DT obtained lower precision records with 79.70% and 98.80% respectively compared to RL + MLP with a score of 99.81% RL + DT with 99.94%.
  - Finally, in [60], although the sample development is more technically oriented, there are no data to compare with ML algorithms to evaluate the quality and shape of the synthetic samples.

It is of utmost importance to mention that the synthetic samples of RL for malware and DoS cyber-attacks were generated from a realistic environment and that, unlike the works presented in the state-of-the-art [67,68,69,70,71,72,73,74] they were not created when the dataset was already characterized or transformed in a tabular, matrix or unstructured way, detaching the observations from the source.

## 6. Conclusions

The area of cybersecurity is dynamic and changing, therefore, malware strains and types of DoS cyber-attacks remain two important points of research, as new, sophisticated and efficient forms of threat grow with greater risk of affectation. The primary objective is to analyze the behavior, origin and causes and thus build sufficiently timely mitigation and remediation mechanisms. Although traditional scopes offer a solution based on SA & DA, the congregation of novel samples have surpassed match & flag mechanisms. At the same time, anomaly-based detection offers a more holistic view that focuses on behavioral analysis, mainly applying ML algorithms, yet obtaining samples can be an arduous and costly process. To overcome this, the generation of synthetic samples using sampling, oversampling, multiple embedding, GANs and data augmentation methods has been proposed. The disadvantage is that such algorithms have presented high computational cost, instability or simply generate identical copies of the original data; in addition, most of the methods are mostly applied when the dataset is already characterized or transformed, detaching from a real and synthetic mutation. This paper presented an RL technique, namely, Reinforsec, that modifies the properties of malware binaries in PE format and DoS cyber-attack data to generate highly functional synthetic samples and thus propose a new and more realistic way to construct datasets for ML purposes. It was demonstrated that the Reinforsec methodology is capable of surpassing the precision levels of many state-of-the-art works.

On the one hand, for the malware scope the RL+DT algorithm outperformed with 99.81% of score others as GAN oriented as GAN + MLP (99.46%), GAN + DT (90.43%), GAN + LR (96.71%), GAN + SVM (97.12%) and GAN + RF (98.87%), DCGAN + ResNet-18 (90.00%) and MDGAN (95.90%). In comparison with MDM-based algorithms, it also obtained better scores in relation: + One-Hot-Encoding + SVM (97.30%), MDM + One-Hot-Encoding + LR (98.10%), Malware MDM + One-Hot-Encoding + RF (98.20%), MDM + AWGCN + SVM (99.20%), MDM + AWGCN + LR (99.30%), and MDM + AWGCN + LR (99.30%) and MDM + AWGCN + RF (98.70%). In this same scope of synthetic malware generation the proposed RL +DT also obtained a better result than its counterpart Fuzzy-SMOTE + SVM (99.02%).

On the side of generating synthetic samples of DoS cyber-attacks, Reinforsec demonstrated with RL + DT that with a precision of 99.94% exceeded Statistical learning + FFNN (88.0), MDM + PRISM + MLP (79.70%) and MDM + PRISM + DT (98.80%) scopes. For the other scopes, we did not obtain enough information and conditions to be able to compare them successfully.

It is worth mentioning that an important contribution of this work is that the samples generated in crude by Reinforsec were subjected to machines such as VirusTotal and CloudShark, which allowed to demonstrate the validity of the samples, giving a broader picture of their functionality.

The future research lines to be addressed are the following: to investigate the behavior of different malware families, in the taxonomic field to learn possible mutations in cryptographic and obfuscation techniques; to propose Reinforsec in the predictive field of malware construction, to understand the logistics of source code and present advances where it is determined how far a malicious software could infect, and, from the technical point of view to observe how to build a simulated attack surface with low computational cost. On the DoS cyber-attack side, we intend to continue working with new types of attacks, low-cost simulations and mutations that are more oriented to the reach of any user who wishes to test raw samples without the need to acquire high-cost stress platforms. An important line of research is volumetric and Distributed Denial of Service (DDoS) attacks, where Reinforsec could get closer to generating realistic changes with different characteristics that resemble those occurring in real time.

Furthermore, as future work, it is being proposed to address synthetic generation focused on the classification of network threats targeting cloud and IoT devices. More areas of opportunity are expected to be covered towards the analysis of emerging technologies such as Software as a Service (SaaS) and Platform as a Service (PaaS).

## Figures and Tables

**Table 1:** Techniques and algorithms commonly used for the generation of synthetic data.

Algorithm	Advantages	Disadvantages
Oversampling	No loss of dataset integrity.	Exact replicates of the samples with minority class are created, or, those with greater distribution are reduced. This is a generalization risk in ML algorithms, as they can lose the sense of generalization, leading to under- or over-fitting events.
Categorical latent Gaussian process	Gaussian processes are flexible, adaptive and easy to manipulate for the generation of new samples.	Phenomena of low or no dispersion can be observed, as they use all samples and features to predict new synthetic samples. In addition, this method may present defective samples when the set has a high dimension.
Multiple embedding	High-dimensional samples are projected onto lower-dimensional samples, producing a new replication with compositionally rich synthetic content.	Samples from different contexts can be represented as one, removing particular and heterogeneous properties, leading to poor generalization.
Generative Adversarial Networks (GAN)	Its major advantage is that a GAN can obtain a latent representation of the original samples and build a new, augmented and modified version according to its distribution.	A large number of continuous samples are needed to generate synthetic outputs, which increases the complexity of the model.
Data augmentation	It generates new points artificially in the existing data, increasing the amount of information in the sample, its main advantage is that it reduces data collection and labeling	It is difficult to provide the necessary augmentation, in fact if the dataset is biased, the augmented data will be biased as well.

**Table 2:** Dataset features, under the EMBER structure.

Group	Feature	Description	Type
1	General information	Encompasses the file characteristics, such as file size, number of imported and exported functions, debugging section, resources, relocations, signatures and number of symbols.	Object
2	PE Header information	Includes the timestamp, target machine and a series of text strings representing the list of read-only data sections. From the optional header, the target subsystem, DLL library imports, the magic number of the file in text format, the major and minor image version, linker versions, system and subsystem versions, code size and headers are depicted.	Object
3	Imported functions	The address import table is translated in a grammatical way and the list of imported functions for each library is reported. In order to create a useful feature for SL models, the set of 256 unique libraries is used, as with the 1024 unique functions, both as an import sequence.	String
4	Exported functions	The features include a list of exported functions which are represented within the object by a 128-binary hash.	String
5	Section information	This group reports the properties of each section of the PE file, including the name, size, entropy, virtual size and a list of text strings that represent the characteristics of the section.	Object
6	Byte histogram	This group covers 256 integer values, which represent the count of each byte contained in the file.	Integer
7	Histogram of entropy bytes	To represent the entropy of the file, the histogram represents the approximation of the probability distribution p(H,b) of the entropy *H* and series of bytes *b*.	Float
8	String information	Statistical information over printable text strings.	Float

**Table 3:** Properties considered to generate a set of appropriate network characteristics.

Properties	Description	Type
Network ports	It is important to mention that in DoS and Distributed-DoS cyber-attacks there is a certain degree of randomness in the target ports used by the attacker, mainly in the TCP protocol and some others specific to the application layer. A valid variety of ports allows the realism of a synthetic flow to be checked.	Integer
Variety of IP addresses	In a DoS cyber-attack, especially a distributed one, there must be a variety of connections from different source IP addresses.	String
Time to live (TTL)	The lifetime of a network packet varies, depending on the metrics of the different network devices, where the attack fluctuates.	Float
Maximum Segment Size (MSS)	It is the distribution of the segments in the capture file and is related to the structure and sequence of the attack.	Float
Window Size	It allows measuring the behavior of packets in relation to the amount of information that a device can receive in a time series.	Integer
Payloads	In attacks targeting the application layer, volumes of payloads can be observed as high length requests directed to specific ports. These, can be schematized as sequences that can be transformed according to their content and volume.	String

**Table 4:** Properties considered to generate a set of appropriate network characteristics.

Feature	Property	Type
Source Address	Variety of IP addresses	String
Origin Protocol	Origin protocol number	String
Destination Protocol	Destination protocol number	String
Destination Address	Variety of IP addresses	String
Packet ID	TTL	String
Source Node	Variety of IP addresses	String
Destination Node	Variety of IP addresses	String
Packet Size	MSS	String
Squencial Number	Window Size	String
Number of Packets	Window Size	String
Number of bytes	Window Size	String
Packet in	TTL	String
Packet out	TTL	String
Packet Transmition	TTL	String
Packet delay note	TTL	String
Packet Rate	Window Size	String
Byte rate	Window Size	String
Pkt Avg Size	Window Size	String
Utilization	Payloads	String
Packet Delay	MSS	String
Packet send time	MSS	String
Packet reserved time	MSS	String
The first packet Sent	TTL	String
Last packet reserved	TTL	String

**Table 5:** Features selected & extracted using the PCA algorithm.

Feature	Description	Type
Forward packet length mean	Mean size of packet in forward direction	Float
Inter-Arrival total bandwidth	Total time between two packets sent in the backward direction	Float
Bandwidth Inter-Arrival time standard deviation	Standard deviation time between two packets sent in the backward direction	Float
Forward push flags	Number of times the push flag was set in packets travelling in the forward direction	Float
Minimum forward segment size	Minimum segment size observed in the forward direction	Float
Forward packet length standard deviation	Standard deviation size of packet in forward direction	Float

**Table 6:** Radius of detection of synthetic samples before characterization.

Synthetic Sample	Detection Radius
Malware	7801 out of 8000 (97.41%) samples detected by VirusTotal sensor.
DDoS	32,120 out of 50,000 (64.24%) samples detected by CloudShark rules

**Table 7:** Related work on synthetic generation of malware and DoS cyber-attacks samples.

Scope	Algorithm	Description	Type of Mutation
Malware	GAN [67]	A GAN with a black box detector is proposed; the samples are modified by changes in the probabilistic distribution of API32 calls, so that, the SL algorithm can misclassify the sample and bypass the detector, thus demonstrating that there are synthetic results with a high degree of obfuscation.	Modification to Windows API32
Malware	DCGAN [68]	Samples of various malware families are converted into 32x32-dimensional gray-scale images. The Deep-Convolutional-GAN network (DCGAN) uses a generator that modifies the original image, adding noise elements in the distribution and using a discriminator to determine whether the modified image is malware or not. It is shown that several malware synthetic samples can be generated by bypassing the discriminator.	Transformation of samples to images and modification of pixel distribution.
Malware	Fuzzy-SMOTE ${}^{☆}$ [69]	Different samples are analyzed, mainly from the Android operating system, representing vecotrized values of SA, DA and risk lists. Synthetic samples are generated by supersampling minority classes in a fuzzy region, to maximize the degree of belonging to the class in question.	Oversampling from minority to majority class.
Malware	MDGAN [70]	A Multifaceted-Deep-GAN (MDGAN) is used to generate a Gussian random distribution to samples containing values from the header of a malware binary in PE format, further, concatenated with sequences from the operating system APIs. The results demonstrate that it is possible to generate features that the discriminator will evaluate as effective malware.	The distribution of the result of merging characteristics is modified.
Malware	Markov Decision Model (MDM) + Attention Aware Graph Neural Network (AWGCN) [71]	The sequences of API calls are modified using Markov chains and then randomly distributed without replacement. It is shown that it is possible to intervene in sequence calling and generate new samples with sequential distributions similar to those of an original malware binary.	The order of the malware binary API sequences in the operating system.
DoS cyber-attacks	Statistical Learning [60]	Descriptive statistical data are obtained as a function of host, protocol, conversation and specific fields of the network flow. PCAP file information is mutated and copied, inferring which values will be closest to a real sample in relation to previously calculated values and maintaining a certain degree of entropy.	The network flow file in PCAP format is modified.
DoS cyber-attacks	Statistical learning and simulation [72]	A simulated environment is generated using specific Internet if Things (IoT) software and statistical data are calculated in the time windows of the attack rerun: the start time and the duration of the attack, and the percentage of the nodes that go under stress. The values are incorporated into a tabular set that is validated by a Neural Network.	Statistical values of a set already constructed.
DoS cyber-attacks	GP-WGANs [73]	The random uniform distribution of different sets in PCAP format is measured using a Gradient Penalty Wasserstein GAN network (GP-WGAN), so that the synthetic samples resemble the real ones. The generator is in charge of executing the probabilistic changes and a discriminator evaluates the quality of the new synthetic sample. This project mainly focuses on application layer attacks.	Data distribution in PCAP files.
DoS cyber-attacks	MDM + Probabilistic Symbolic Symbolic Model Checker (PRISM) [74]	It focuses on simulating the steps to synthetically reproduce a DDoS attack on an IoT sensor network, thanks to the transactional abstractions of the MDM. PRISM allows to calculate the probability of sensor battery drain, specifically in application layer attacks, allowing to generate data that evaluate the intensity of a volumetric attack.	Attack sequences and probability of battery drainage.

${}^{☆}$ SMOTE stands for Synthetic Minority Oversampling Technique.

**Table 8:** State-of-the-art reported SL and DL algorithm configurations for comparison with this work (RL).

Algorithm	Configuration
	Hidden layer sizes: $H_{1},H_{2}\in R^{32}$
RL + MLP	Activation function: sigmoid
	Weight optimization solver: Stochastic Gradient Descent
	Attribute selection method: GINI ${}^{§}$
RL + RF	Number of features to consider for best split: 2
	Minimum number of samples required to be at leaf node: 1
	Minimum number of samples required to split internal nodes: 1
	Maximum depth of the tree: 3
	Minimum number of trees in forest: 3
	Number of estimators (trees): 100
RL + DT	Maximum number of features in each estimator: 3
	Maximum depth of the tree: 3
	Inverse of regularization strength of term: 1.0
RL + LR	Norm selected to regularize the cost function: $\ell_{2}$
	Optimization algorithm: LBFGS ${}^{‡}$
	Penalty parameter *C* of error term: 10
RL + SVM	Type of division: One-vs-one
	Kernel type: linear
	Loss function to be optimized: log-loss
RL + GB	Number of estimators: 100
	Criterion to measure the quality of a split: Friedman Minimum-Square-Error
	Minimum number of samples required to split internal nodes: 2
	Minimum number of samples required to be at leaf node: 1
	1D convolution layer *L* with 64 filters, a kernel size with 3 units and as an activation function ReLU
	1×[7×7] convolution layer
	$4\times [3\times 3\in R^{64}$ convolution layer]
RL + ResNET-18	$4\times [3\times 3\in R^{128}$ convolution layer]
	$4\times [3\times 3\in R^{256}$ convolution layer]
	$4\times [3\times 3\in R^{512}$ convolution layer]
	The output layer O∈R with a Sigmoid activation function
	1D Dense input layer *L* with 9 units and as an activation function ReLU
RL + FFNN	1 Hidden layer $H_{1}\in R^{256}$
	1 Hidden layer $H_{2}\in R^{128}$
	The output layer O∈R with a Sigmoid activation function

${}^{§}$ stands for Entropy and Information Gain and ${}^{‡}$ for Limited-memory Broyden–Fletcher–Goldfarb–Shannon algorithm.

**Table 9:** State-of-the-art reported SL and DL algorithm results for comparison with this work (RL).

Scope	Algorithm	Precision
	GAN + MLP [67]	99.46 %
	GAN + DT [67]	90.43%
Malware	GAN + LR [67]	96.71%
	GAN + SVM [67]	97.12%
	GAN + RF [67]	98.87%
Malware	DCGAN + ResNet-18 [68]	90.00%
Malware	Fuzzy-SMOTE + SVM [69]	99.02%
Malware	MDGAN [70]	95.90%
	MDM + One-Hot-Encoding + SVM [71]	97.30%
	MDM + One-Hot-Encoding + LR [71]	98.10%
Malware	MDM + One-Hot-Encoding + RF [71]	98.20%
	MDM + AWGCN + SVM [71]	99.20%
	MDM + AWGCN + LR [71]	99.30%
	MDM + AWGCN + RF [71]	98.70%
	RL + MLP (this work)	99.12%
	RL + DT (this work)	99.71%
	RL + LR (this work)	99.45%
Malware	RL + SVM (this work)	98.70%
	RL + RF (this work)	99.81%
	RL + ResNet-18 (this work)	92.54%
DoS cyber-attacks	Statistical learning + FFNN [72]	88.00%
DoS cyber-attacks	GP-WGANs + GB [73]	AUC = 0.75
DoS cyber-attacks	Statistical Learning [60]	-
	MDM + PRISM + MLP [74]	79.70%
DoS cyber-attacks	MDM + PRISM + DT [74]	98.80%
	RL + MLP (this work)	99.81%
DoS cyber-attacks	RL + DT (this work)	99.94%
	RL + GB (this work)	AUC = 0.87
	RL + FFNN (this work)	96.41%

## Data Availability

Not applicable.

## References

[1] (2021). Enisa Threat Landscape 2021. https://www.enisa.europa.eu/publications/enisa-threat-landscape-2021.

[2] Kolias C., Kambourakis G., Stavrou A., Voas J. (2017). DDoS in the IoT: Mirai and other botnets. Computer.

[3] Moore T. (2010). The economics of cybersecurity: Principles and policy options. Int. J. Crit. Infrastruct..

[4] Leszczyna R. (2021). Review of cybersecurity assessment methods: Applicability perspective. Comput. Secur..

[5] Ford V., Siraj A. (2014). Applications of machine learning in cyber security. Proceedings of the 27th International Conference on Computer Applications in Industry and Engineering.

[6] Ucci D., Aniello L., Baldoni R. (2019). Survey of machine learning techniques for malware analysis. Comput. Secur..

[7] (2019). McAfee Labs and Advanced Threat Research. McAfee Labs Threats Report. https://www.trellix.com/fr-ca/advanced-research-center/threat-reports.html.

[8] Yu B., Fang Y., Yang Q., Tang Y., Liu L. (2018). A survey of malware behavior description and analysis. Front. Inf. Technol. Electron..

[9] Khalaf B.A., Mostafa S.A., Mustapha A., Mohammed M.A., Abduallah W.M. (2019). Comprehensive review of artificial intelligence and statistical approaches in distributed denial of service attack and defense methods. IEEE Access.

[10] Valdovinos I.A., Perez-Diaz J.A., Choo K.K.R., Botero J.F. (2021). Emerging DDoS attack detection and mitigation strategies in software-defined networks: Taxonomy, challenges and future directions. J. Netw. Comput. Appl..

[11] Nikoloudakis Y., Kefaloukos I., Klados S., Panagiotakis S., Pallis E., Skianis C., Markakis E.K. (2021). Towards a Machine Learning Based Situational Awareness Framework for Cybersecurity: An SDN Implementation. Sensors.

[12] Handa A., Sharma A., Shukla S.K. (2019). Machine learning in cybersecurity: A review. Wiley Interdiscip. Rev. Data Min. Knowl. Discov..

[13] Shaukat K., Luo S., Varadharajan V., Hameed I.A., Xu M. (2020). A survey on machine learning techniques for cyber security in the last decade. IEEE Access.

[14] Roh Y., Heo G., Whang S.E. (2019). A survey on data collection for machine learning: A big data-ai integration perspective. IEEE Trans. Knowl. Data Eng..

[15] Paullada A., Raji I.D., Bender E.M., Denton E., Hanna A. (2021). Data and its (dis) contents: A survey of dataset development and use in machine learning research. Patterns.

[16] Sarker I.H., Kayes A., Badsha S., Alqahtani H., Watters P., Ng A. (2020). Cybersecurity data science: An overview from machine learning perspective. J. Big Data.

[17] Humayun M., Jhanjhi N., Talib M., Shah M.H., Suseendran G. (2021). Cybersecurity for Data Science: Issues, Opportunities, and Challenges. Lect. Notes Netw. Syst..

[18] Alshaibi A., Al-Ani M., Al-Azzawi A., Konev A., Shelupanov A. (2022). The Comparison of Cybersecurity Datasets. Data.

[19] Dasgupta D., Akhtar Z., Sen S. (2022). Machine learning in cybersecurity: A comprehensive survey. J. Def. Model. Simul..

[20] Sarker I.H. (2019). A machine learning based robust prediction model for real-life mobile phone data. Internet Things..

[21] Zheng M., Robbins H., Chai Z., Thapa P., Moore T. Cybersecurity research datasets: Taxonomy and empirical analysis. Proceedings of the 11th USENIX Workshop on Cyber Security Experimentation and Test (CSET 18).

[22] Naseer M., Rusdi J.F., Shanono N.M., Salam S., Muslim Z.B., Abu N.A., Abadi I. (2021). Malware Detection: Issues and Challenges. Proceedings of the 2019 International Conference of Science and Information Technology in Smart Administration (ICSINTeSA).

[23] Alzahrani R.J., Alzahrani A. (2021). Security Analysis of DDoS Attacks Using Machine Learning Algorithms in Networks Traffic. Electronics.

[24] Sikorsi A.M. (2012). Practical Malware Analysis: A Hands-On Guide to Dissecting Malicious Software.

[25] Nikolenko S.I. (2021). Synthetic Data for Deep Learning.

[26] Ye J., Xue Y., Long L.R., Antani S., Xue Z., Cheng K.C., Huang X. Synthetic sample selection via reinforcement learning. Proceedings of the International Conference on Medical Image Computing and Computer-Assisted Intervention.

[27] Polizzotto M.N., Finfer S., Garcia F., Sönnerborg A., Zazzi M., Böhm M., Jorm L., Barbieri S., Kaiser R., I-Hsien Kuo N. (2022). The Health Gym: Synthetic Health-Related Datasets for the Development of Reinforcement Learning Algorithms. arXiv.

[28] Arulkumaran K., Deisenroth M.P., Brundage M., Bharath A.A. (2017). Deep reinforcement learning: A brief survey. IEEE Signal Process. Mag..

[29] Brockman G., Cheung V., Pettersson L., Schneider J., Schulman J., Tang J., Zaremba W. (2016). Openai gym. arXiv.

[30] Xiang X., Foo S. (2021). Recent Advances in Deep Reinforcement Learning Applications for Solving Partially Observable Markov Decision Processes (POMDP) Problems: Part 1—Fundamentals and Applications in Games, Robotics and Natural Language Processing. Mach. Learn. Knowl. Extr..

[31] Sutton R.S., Barto A.G. (2018). Reinforcement Learning: An Introduction.

[32] Singh J., Singh J. (2021). A survey on machine learning-based malware detection in executable files. J. Syst. Archit..

[33] Aboaoja F.A., Zainal A., Ghaleb F.A., Al-rimy B.A.S., Eisa T.A.E., Elnour A.A.H. (2022). Malware Detection Issues, Challenges, and Future Directions: A Survey. Appl. Sci..

[34] Karl-Bridge-Microsoft. PE Format-Win32 Apps. https://github.com/Karl-Bridge-Microsoft.

[35] Zatloukal F., Znoj J. (2017). Malware detection based on multiple PE headers identification and optimization for specific types of files. JAEC.

[36] Anderson H.S., Kharkar A., Filar B., Evans D., Roth P. (2018). Learning to Evade Static PE Machine Learning Malware Models via Reinforcement Learning. arXiv.

[37] Salem A., Banescu S., Pretschner A. (2021). Maat: Automatically analyzing virustotal for accurate labeling and effective malware detection. ACM Trans. Priv. Secur..

[38] (2021). VirusTotal. Virustotal.

[39] Zhao Y., Li L., Wang H., Cai H., Bissyandé T.F., Klein J., Grundy J. (2021). On the impact of sample duplication in machine-learning-based android malware detection. ACM Trans. Softw. Eng. Methodol..

[40] Joyce R.J., Amlani D., Nicholas C., Raff E. (2022). MOTIF: A Malware Reference Dataset with Ground Truth Family Labels. Comput. Secur..

[41] Oyama Y., Miyashita T., Kokubo H. Identifying useful features for malware detection in the ember dataset. Proceedings of the 2019 Seventh International Symposium on Computing and Networking Workshops (CANDARW).

[42] Amich A., Eshete B. Explanation-guided diagnosis of machine learning evasion attacks. Proceedings of the International Conference on Security and Privacy in Communication Systems.

[43] Castro R.L., Schmitt C., Rodosek G.D. Armed: How automatic malware modifications can evade static detection?. Proceedings of the 2019 5th International Conference on Information Management (ICIM).

[44] Romain T. LIEF Library to Instrument Executable Formats. https://lief-project.github.io/.

[45] Anderson H.S., Roth P. (2018). Ember: An open dataset for training static pe malware machine learning models. arXiv.

[46] Hawkins D.M. (2004). The problem of overfitting. J. Chem. Inf. Model.

[47] Weinberger K., Dasgupta A., Langford J., Smola A., Attenberg J. Feature hashing for large scale multitask learning. Proceedings of the 26th Annual International Conference on Machine Learning.

[48] Vishnu N., Batth R.S., Singh G. Denial of service: Types, techniques, defence mechanisms and safe guards. Proceedings of the 2019 International Conference on Computational Intelligence and Knowledge Economy (ICCIKE).

[49] Pokrinchak M., Chowdhury M.M. Distributed Denial of Service: Problems and Solutions. Proceedings of the 2021 IEEE International Conference on Electro Information Technology (EIT).

[50] Bhardwaj A., Mangat V., Vig R., Halder S., Conti M. (2021). Distributed denial of service attacks in cloud: State-of-the-art of scientific and commercial solutions. Comput. Sci. Rev..

[51] Shinde P., Parvat T.J. (2016). DDoS attack analyzer: Using JPCAP and WinCap. Procedia Comput. Sci..

[52] Goyal P., Goyal A. Comparative study of two most popular packet sniffing tools-Tcpdump and Wireshark. Proceedings of the 2017 9th International Conference on Computational Intelligence and Communication Networks (CICN).

[53] Kshirsagar D., Kumar S. (2022). A feature reduction based reflected and exploited DDoS attacks detection system. JAIHC.

[54] Arshi M., Nasreen M., Madhavi K. (2020). A survey of DDoS attacks using machine learning techniques. Proceedings of the E3S Web of Conferences.

[55] Zargar S.T., Joshi J., Tipper D. (2013). A survey of defense mechanisms against distributed denial of service (DDoS) flooding attacks. IEEE Commun. Surv. Tutor..

[56] Gohil M., Kumar S. Evaluation of classification algorithms for distributed denial of service attack detection. Proceedings of the 2020 IEEE Third International Conference on Artificial Intelligence and Knowledge Engineering (AIKE).

[57] Kaspersky (2015). DDoS Protection White Paper.

[58] Sharafaldin I., Lashkari A.H., Hakak S., Ghorbani A.A. Developing realistic distributed denial of service (DDoS) attack dataset and taxonomy. Proceedings of the 2019 International Carnahan Conference on Security Technology (ICCST).

[59] Radoyska P., Atanasova M. Free tools for Testing the Security of Web Services in the UTP Network. Proceedings of the Fifth International Scientific Conference “Telecommunications, Informatics, Energy and Management”.

[60] Cordero C.G., Vasilomanolakis E., Wainakh A., Mühlhäuser M., Nadjm-Tehrani S. (2021). On generating network traffic datasets with synthetic attacks for intrusion detection. ACM Trans. Priv. Secur..

[61] Alkasassbeh M., Al-Naymat G., Hassanat A.B., Almseidin M. (2016). Detecting distributed denial of service attacks using data mining techniques. Int. J. Adv. Comput. Sci. Appl..

[62] Alothman B. Raw network traffic data preprocessing and preparation for automatic analysis. Proceedings of the 2019 International Conference on Cyber Security and Protection of Digital Services (Cyber Security).

[63] Han L.q., Zhang Y. Pca-based ddos attack detection of sdn environments. Proceedings of the International conference on Big Data Analytics for Cyber-Physical-Systems.

[64] Bro R., Smilde A.K. (2014). Principal component analysis. Anal. methods.

[65] Masri R., Aldwairi M. Automated malicious advertisement detection using virustotal, urlvoid, and trendmicro. Proceedings of the 2017 8th International Conference on Information and Communication Systems (ICICS).

[66] Sanders C. (2017). Practical Packet Analysis, 3E: Using Wireshark to Solve Real-World Network Problems.

[67] Zhu E., Zhang J., Yan J., Chen K., Gao C. (2022). N-gram MalGAN: Evading machine learning detection via feature n-gram. Digit. Commun. Netw..

[68] Lu Y., Li J. Generative adversarial network for improving deep learning based malware classification. Proceedings of the 2019 Winter Simulation Conference (WSC).

[69] Xu Y., Wu C., Zheng K., Niu X., Yang Y. (2017). Fuzzy–synthetic minority oversampling technique: Oversampling based on fuzzy set theory for Android malware detection in imbalanced datasets. Int. J. Distrib. Sens. Netw..

[70] Mazaed Alotaibi F. (2022). A Multifaceted Deep Generative Adversarial Networks Model for Mobile Malware Detection. Appl. Sci..

[71] Hsiao S.W., Chu P.Y. (2022). Sequence Feature Extraction for Malware Family Analysis via Graph Neural Network. arXiv.

[72] Hekmati A., Grippo E., Krishnamachari B. Large-scale Urban IoT Activity Data for DDoS Attack Emulation. Proceedings of the 19th ACM Conference on Embedded Networked Sensor Systems.

[73] Charlier J., Singh A., Ormazabal G., State R., Schulzrinne H. (2019). SynGAN: Towards generating synthetic network attacks using GANs. arXiv.

[74] Arnaboldi L., Morisset C. Generating synthetic data for real world detection of DoS attacks in the IoT. Proceedings of the Software Technologies: Applications and Foundations.

[75] Hernandez-Suarez A., Sanchez-Perez G., Toscano-Medina L.K., Olivares-Mercado J., Portillo-Portilo J., Avalos J.G., García Villalba L.J. (2022). Detecting Cryptojacking Web Threats: An Approach with Autoencoders and Deep Dense Neural Networks. Appl. Sci..

[76] Liu M., Mroueh Y., Ross J., Zhang W., Cui X., Das P., Yang T. (2019). Towards better understanding of adaptive gradient algorithms in generative adversarial nets. arXiv.

